# Advanced bioartificial organs: genetically modified pig liver as a promising bridge for human liver failure

**DOI:** 10.1038/s41392-025-02276-x

**Published:** 2025-06-02

**Authors:** Taeho Kwon, Sun-Uk Kim, Kyungjun Uh

**Affiliations:** 1 Primate Resources Center, Korea Research Institute of Bioscience and Biotechnology (KRIBB), Jeongeup, Jeonbuk Republic of Korea; 2 Futuristic Animal Resource and Research Center, Korea Research Institute of Bioscience and Biotechnology (KRIBB), Cheongju, Chungbuk Republic of Korea; 3 Advanced Bioconvergence Department, Department of Functional Genomics, KRIBB School, Korea National University of Science and Technology (UST), Daejeon, Republic of Korea

**Keywords:** Biotechnology, Medical research

In a groundbreaking study recently published in *Nature*, Kai-Shan Tao and colleagues achieved the first successful heterotopic pig-to-human liver xenotransplantation using a gene-edited Bama miniature pig carrying six targeted genetic modifications designed to suppress immunologic rejection and thrombosis.^1^ The ability of the graft to maintain bile secretion, albumin synthesis, and histological integrity over 10 days in a brain-dead human recipient underscores the potential of xenotransplantation as a viable solution to the global shortage of donor livers for patients with acute or end-stage liver failure.^1^

Central to the success of this approach was the precise engineering of the donor pig’s genome. Researchers eliminated three major xenoantigen genes (GGTA1, CMAH, and B4GALNT2) encoding glycan epitopes widely recognized by human natural antibodies, effectively preventing hyperacute rejection. Simultaneously, three human transgenes (CD46, CD55, and hTHBD) were integrated to enhance complement regulation and minimize thrombosis. These combined modifications created a safer immunological and hemodynamic environment for the xenograft.

**Fig. 1:**
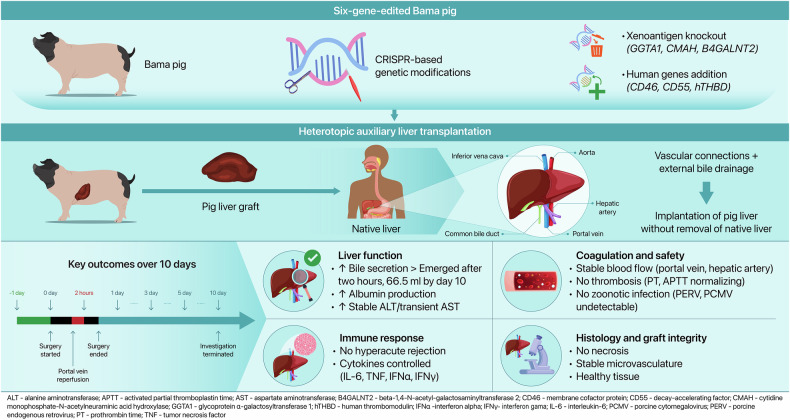
Genetically modified pig liver xenograft in a human recipient. A Bama miniature pig was genetically engineered with six specific modifications, including three Xenoantigen knockouts (GGTA1, CMAH, B4GALNT2) and three Human transgenes (CD46, CD55, hTHBD). The liver was transplanted heterotopically into a brain-dead human recipient while retaining the native liver. Over a 10-day observation period, the graft demonstrated bile secretion and porcine albumin production, with no signs of hyperacute rejection or thrombosis. Histological examination confirmed intact liver architecture, and no zoonotic viruses were detected. These findings support the feasibility of using gene-edited porcine livers as temporary hepatic support systems in humans. This figure was created using Editage (www.editage.com)

The clinical protocol featured a heterotopic auxiliary liver transplantation technique, in which the porcine liver was implanted without removing the native human liver. This allowed careful monitoring of graft performance while minimizing surgical risks. Preoperative imaging ensured alignment of the porcine portal vein and hepatic artery with the recipient’s vasculature, guaranteeing functional blood flow. Postoperatively, bile was diverted externally for direct measurement of secretory capacity. Within just 2 h of reperfusion, the graft began secreting bile—a reliable early indicator of hepatic viability. Throughout the 10-day period, the graft continuously secreted bile, indicating preserved exocrine capability (Fig. 1).

Concurrently, metabolic activity was demonstrated through steadily increasing porcine-derived albumin levels in the recipient’s circulation, confirming active hepatic protein synthesis. Serum biomarkers of liver injury remained largely stable, aside from a transient spike in aspartate aminotransferase (AST) shortly after transplantation. The authors suggested that the AST elevation could be cardiac in origin, partly supported by simultaneous rises in cardiac enzyme markers. Later increases in bilirubin and γ-glutamyl transpeptidase likely mirrored mild cholestatic changes in the native liver rather than the xenograft. These findings collectively indicated that the pig liver maintained structural and metabolic integrity without pronounced hepatocellular damage.

Detailed histopathological analyses corroborated the functional data. Graft biopsy samples showed intact lobular architecture, minimal sinusoidal congestion, and no significant necrosis or inflammatory infiltrates. Immunohistochemistry revealed low-level complement deposition (C3d, C4d, C5b-9) and only sparse immune cell presence, indicating effective suppression of major rejection pathways.^1,2^ Markers of tissue regeneration and vascular integrity indicated healthy cellular turnover and stable microvasculature. Transmission electron microscopy further underscored the absence of ultrastructural damage or viral inclusions, an important milestone for xenotransplant safety.^2^

Hyperacute rejection remains a major barrier in xenotransplantation, triggered by human antibodies binding to pig antigens, activating complement, causing thrombosis, and graft failure.^2^ By eliminating key xenoantigen genes and embedding human complement regulatory genes, the team drastically reduced these immunologic triggers. Additionally, an immunosuppressive regimen optimized for xenotransplantation reinforced graft stability, including anti-thymocyte globulin for T-cell depletion, eculizumab for complement inhibition, and corticosteroids, tacrolimus, and mycophenolate mofetil for maintenance therapy. B cell counts, initially rising, were effectively managed by administering rituximab. Cytokine (IL-6, TNF, IFN-α, IFN-γ) and inflammatory markers (CRP, procalcitonin) remained controlled, reflecting effective immunomodulation.

Preventing thrombotic complications was another critical achievement. Incorporating human thrombomodulin (hTHBD) and complement regulatory proteins minimized prothrombotic stimuli. Post-transplant Doppler ultrasound monitoring confirmed stable blood flow in graft vessels. Platelet counts, initially decreased, rebounded by the study’s end. Coagulation profiles were largely normal aside from a temporary increase in activated partial thromboplastin time (APTT). Early increase in D-dimer was successfully managed with thrombolytics, preventing significant clot formation.

Safety also involved mitigating zoonotic risks, particularly porcine endogenous retrovirus (PERV) and porcine cytomegalovirus (PCMV). Neither pathogen was detected in the recipient’s tissues or circulation. No evidence of microchimerism was observed, and the recipient’s inferior vena cava was successfully reconstructed following graft removal, with no observed complications, which demonstrated the feasibility of short-term xenograft support. In preparation for clinical application, xenotransplantation safety systems must evolve beyond pathogen detection. Current efforts include PERV inactivation, designated pathogen-free (DPF) breeding conditions, and high-sensitivity microbial surveillance. Moving forward, robust systems integrating real-time viral monitoring, zoonotic risk assays, long-term recipient follow-up, and stringent environmental controls will be essential.^2^ The incorporation of ethical oversight and regulatory alignment will further support safe clinical translation. To minimize the risk of cross-species infection during clinical translation, xenograft programs incorporate multilayered microbial safety strategies. These include maintaining donor herds under DPF conditions, utilizing next-generation sequencing to detect latent or emerging zoonotic viruses, and implementing stringent environmental and recipient surveillance protocols. Such systems are essential for detecting and intercepting potential infectious threats before clinical manifestation.

Despite these promising results, the study has several limitations, including the short observation period and the use of a brain-dead recipient, which restricts the ability to draw conclusions about long-term graft function, neuroendocrine integration, and overall patient recovery. Furthermore, while bile secretion and albumin synthesis are important markers of liver function, they do not fully capture the organ’s comprehensive metabolic and detoxification capacity. Evaluation of parameters such as ammonia clearance, hormonal regulation, and energy metabolism would provide a deeper understanding of xenogeneic graft performance. Long-term studies in living human recipients will be essential to assess chronic immunosuppression, graft durability, and the psychological implications of receiving an organ from another species.^2,3^ Although the current study focuses primarily on physiological outcomes, future investigations should clarify the molecular signaling pathways influenced by specific gene-editing targets, such as CD46 in complement regulation and hTHBD in coagulation control, as well as their downstream effects on endothelial integrity and immune activation. Deeper mechanistic insight into these pathways could inform the refinement of gene-editing approaches and the development of targeted immunotherapy strategies to optimize clinical xenograft performance. Nevertheless, this work represents a significant advancement toward clinical xenotransplantation. The demonstration of stable function and minimal complications in a genetically modified pig liver underscores substantial progress achieved through CRISPR-based genome editing, refined surgical techniques, and targeted immunosuppressive regimens.^4^ Ongoing efforts involving pig kidneys and hearts further support the emergence of multi-organ xenotransplantation as a frontier in transplant medicine.^4,5^ Ultimately, auxiliary liver grafts may offer critical support for patients with acute liver failure while they await recovery or a suitable human donor.^1,5^

Looking ahead, integrating synthetic biology, regenerative medicine, and precision immunology may further enhance clinical use of porcine organs—through optimized glycan editing, protective transgenes, and personalized immunosuppression protocols. Vigilant monitoring for zoonoses and malignancies remains essential. Nonetheless, in light of the persistent global organ shortage, the study by Tao et al. marks a critical step toward making xenotransplantation clinically viable. If replicated in living human recipients, gene-edited pig organs may soon become a viable therapeutic option in transplantation medicine.^1,5^ However, the safe and effective clinical translation of this strategy will require systematic evaluation of immunologic, surgical, and physiological complexities. Although the present study offers functional validation of xenogeneic liver support in a human recipient, further progress toward clinical application will depend on effectively addressing persistent challenges in both auxiliary and orthotopic transplant settings. These include ensuring immunological compatibility, refining surgical techniques for vascular anastomosis, and overcoming limitations in replicating the liver’s complex physiological roles in preclinical in vitro and in vivo models.
